# Tourniquet use following blast-associated complex lower limb injury and traumatic amputation promotes end organ dysfunction and amplified heterotopic ossification formation

**DOI:** 10.1186/s13018-022-03321-z

**Published:** 2022-09-19

**Authors:** Philip J. Spreadborough, Amy L. Strong, John Mares, Benjamin Levi, Thomas A. Davis

**Affiliations:** 1 Department of Surgery, Uniformed Services University of the Health Sciences, 4301 Jones Bridge Road, Bethesda, MD 20814 USA; 2 Academic Department of Military Surgery and Trauma, Royal Centre for Defence Medicine, Birmingham, UK; 3 Division of Plastic and Reconstructive Surgery, Department of Surgery, University of Michigan, Ann Arbor, MI USA; 4 Department of Surgery, University of Texas Southwestern Medical Center, Dallas, TX USA

**Keywords:** Tourniquets, Blast injury, Traumatic heterotopic ossification, Complex limb injury, Ischemia reperfusion

## Abstract

**Background:**

Traumatic heterotopic ossification (tHO) is characterized by ectopic bone formation in extra-skeletal sites leading to impaired wound healing, entrapment of neurovascular structures, pain, and reduced range of motion. HO has become a signature pathology affecting wounded military personnel who have sustained blast-associated traumatic amputations during the recent conflicts in Iraq and Afghanistan and can compound recovery by causing difficulty with prosthesis limb wearing. Tourniquet use to control catastrophic limb hemorrhage prior to surgery has become almost ubiquitous during this time, with the recognition the prolonged use may risk an ischemia reperfusion injury and associated complications. While many factors influence the formation of tHO, the extended use of tourniquets to limit catastrophic hemorrhage during prolonged field care has not been explored.

**Methods:**

Utilizing an established pre-clinical model of blast-associated complex lower limb injury and traumatic amputation, we evaluated the effects of tourniquet use on tHO formation. Adult male rats were subjected to blast overpressure exposure, femur fracture, and soft tissue crush injury. Pneumatic tourniquet (250–300 mmHg) applied proximal to the injured limb for 150-min was compared to a control group without tourniquet, before a trans-femoral amputation was performed. Outcome measures were volume to tHO formation at 12 weeks and changes in proteomic and genomic markers of early tHO formation between groups.

**Results:**

At 12 weeks, volumetric analysis with microCT imaging revealed a 70% increase in total bone formation (*p* = 0.007) near the site of injury compared to rats with no tourniquet time in the setting of blast-injuries. Rats subjected to tourniquet usage had increased expression of danger-associated molecular patterns (DAMPs) and end organ damage as early as 6 h and as late as 7 days post injury. The expressions of pro-inflammatory cytokines and chemokines and osteochondrogenic genes using quantitative RT-PCR similarly revealed increased expression as early as 6 h post injury, and these genes along with hypoxia associated genes remained elevated for 7 days compared to no tourniquet use.

**Conclusion:**

These findings suggest that tourniquet induced ischemia leads to significant increases in key transcription factors associated with early endochondral bone formation, systemic inflammatory and hypoxia, resulting in increased HO formation.

## Background

Traumatic heterotopic ossification (tHO) has become a signature pathology affecting wounded military personnel sustaining blast-associated traumatic amputations during recent conflicts in Iraq and Afghanistan [1, 2]. Additionally, it is a common complication seen after high-energy civilian trauma, large surface area burns and hip arthroplasty surgery, where therapies such as radiotherapy and non-steroidal anti-inflammatory medications (NSAIDs) have been used to reduce HO formation [3–7]. Traumatic HO is characterized by the abnormal development of mature lamellar bone in extra-skeletal sites, such as muscle, tendons and other soft tissues, which can lead to impaired wound healing, entrapment of neurovascular structures, increased pain, reduced range of motion, and difficulty with prosthetic applications [1, 8]. It is estimated that up to 90% of blast-associated traumatic amputations will develop evidence of heterotopic ossification (HO) [2, 9], with blast exposure and amputation through the zone of injury (ZOI) being factors associated with both HO development and severity [2, 10].

Blast-associated traumatic amputations are often associated with catastrophic hemorrhage, leading to the use of tourniquets on limbs to control extremity bleeding [11–18]. While tourniquet use controls hemorrhage, this is at the expense of inducing global limb ischemia distal to the level of tourniquet application, with prolonged tourniquet use being associated with complications, such as neuropraxia, muscle fibrosis and necrosis, compartment syndrome, limb loss, and mortality [19–21]. Previous studies have often shown durations of greater than 2 h of tourniquet application to be associated with greater complications and metabolic insults [11, 22]. Exhaustion of intracellular energy stores and predisposition to muscle fiber damage, necrosis, and microvascular injury has been shown to occur after two hours of ischemia. Post-revascularization reperfusion following prolonged ischemia has also been shown to trigger the activation of the inflammatory cascade, resulting in the release of cytokines and chemokines, which if dysregulated leads to further damage to cells causing multiple secondary end organ dysfunction and morbidity [23, 24]. Thus, less than two hours of ischemia time is often used as the advised maximum duration of tourniquet application to prevent the risk of ischemia reperfusion injury (IRI). However, during recent military conflicts, limited tourniquet duration may be logistically challenging and unachievable. Additionally, in civilian complex extremity trauma cases, prolonged tourniquet use is often required, and prolonged cases of ischemia may occur while trying to reestablish perfusion surgically. Therefore, there is a need to understand the role of tourniquet use and the development of IRI in combat-injured limbs to limit the formation of tHO. While the biological basis for a correlation between tourniquet use in the combat-injured limbs and the development of HO has been theorized [25], the precise mechanism remains to be determined.

Hypoxia appears to play a vital role in normal skeletal development and aberrant wound healing, involving the highly orchestrated interactions between different cells and signaling pathways to form mineralized tissue [26]. Furthermore, aberrant hypoxia signaling in injured muscle and soft tissue has been shown to be a common link between both tHO and genetic forms of HO, such as fibrodysplasia ossificans progressiva [27, 28]. The main hypoxia/oxygen-sensing signaling molecule that controls oxygen responsive genes is hypoxia-inducible factor-1 alpha (HIF-1α). HIF-1α is the master transcription factor regulating diverse cellular and systemic responses to tissue hypoxia and is a critical signaling mediator for osteochondrogenic differentiation [29, 30]. Hypoxia-dependent upregulation of HIF-1α affects many target genes including vascular endothelial growth factor (VEGF), angiopoietin-1 (ANGPT)/TIE-2, and vascular endothelial growth factor receptor-1 (VEGFR-1)/FLT1 signaling that influences the secretion of inflammatory cytokines and growth factors, the migration and proliferation of endothelial cells and fibroblasts, cellular metabolism, energy consumption, cellular apoptosis, leukocyte adhesion, and tissue repair-wound healing. The association between tissue hypoxia following ischemic injury, HIF-1α upregulation, angiogenesis and subsequent osteogenesis presents a mechanism by which tissue hypoxia could increase HO formation. Therefore, we sought to address the hypothesis that prolonged tourniquet use exacerbates tHO through the upregulation of hypoxia-inducible pathways which results in greater angiogenic and osteogenic signaling. Using our established blast combat applicable model of traumatic lower limb amputation, we assessed the impact of tourniquet-induced skeletal muscle IRI on remote organ function and tHO formation.

## Methods

### Animals

Adult male (11–12-week-old (350–450 gm) specific pathogen free Sprague Dawley rats (*Rattus norvegicus*) were obtained from Taconic Biosciences (Germantown, NY). Animals were housed for a minimum of 7 days for acclimatization and quarantine purposes. All rats were housed in clean clear plastic cages and exposed to a 12-h light/dark cycle, with free access to food (standard rodent chow) and water, under veterinary care and supervision. All experiments and animal care procedures for this research were approved by the Naval Medical Research Center (NMRC), Walter Reed Army Institute of Research (WRAIR), and the Uniformed Services University Institutional Animal and Care and Use Committees (IACUC). All activities were conducted in accordance with all applicable regulations and best practices pertaining to the use of animals in research.

### Blast associated complex lower limb injury model

Rats were subjected to the well-established blast-associated complex lower limb injury model, as previously described [31–34]. Briefly, following induction of anesthesia and analgesia administration, rats were secured into a holding device in the pneumatically driven shock tube apparatus and were exposed to a peak overpressure of 120 ± 7 kPa [35–37]. A custom lateral long bone ballistic system [38, 39] utilizing a drop weight of 581 g from a height of 88 cm created a femoral diaphysis fracture, followed immediately post fracture by a crush injury with a force of 138 kPa (20 psi), calibrated against a digital force gauge (DFE II, AMETEK, FL), for 1 min. Based on group allocation, rats were transferred to the thermoregulated heating blanket for tourniquet application of 150 min (Blast Complex HLI 150), while the control group underwent immediate amputation (Blast Complex Control). Hind limb ischemia was achieved by the application of a pneumatic tourniquet (UDC1.6™, Hokanson, WA) to the proximal aspect of the limb [40–42]. Elevation of the limb prior to tourniquet application was performed to reduce venous pooling, reducing the likelihood of thrombotic complications following tourniquet release. Tourniquets were inflated and controlled using a segmental cuff selector enabling rapid and independent inflation and deflation (MV10, Hokanson, WA), connected to an aneroid sphygmomanometer (DS400, Hokanson, WA). Inflation pressures were maintained at 280–300 mmHg for 150 min, which exceeded the 220 mmHg, which has been previously reported to stop microvascular flow in a rat limb [40, 41]. Immediately following tourniquet-induced ischemia, trans-femoral amputation was performed through the zone of injury (ZOI) using a separate sterile field and dissection kit for each animal. Closure was completed in a layered fashion. All rats received anesthesia and/or analgesia prior to any procedures (blast exposure, tourniquet application, surgical procedures) and after 48–72 h following the surgical procedure to reduce any discomfort experienced and to minimize confounders from stress.

### In vivo micro computed tomography (μCT)

In vivo μCT imaging was performed as previously described using a SkyScan 1176 in vivo high-resolution μCT (Bruker, Kontrich, Belgium) [32, 33]. Briefly, imaging was conducted at post-operative days (POD) 14, 28, 56 and 84. Scans were conducted with the same settings as previous studies; 89-kV polychromatic x-ray beam, 256 μA current and an exposure time of 81 ms per 180° rotation. Images were processed using NRecon Reconstruction software (Bruker, Kontrich, Belgium) to align scan images and generate reconstructed images and cross-sectional images.

### Euthanasia and tissue collection and storage

Study end timepoints were at day 84 in the observational study (*n* = 14 per group) assessing tHO formation, and at 6 h, 24 h, post-operative day (POD) 3, or POD 7 in study groups used to assess genomic and proteomic changes (*n* = 7–8 per group per timepoint). Rats were euthanized in accordance with the 2013 American Veterinary Medical Association (AVMA) Guidelines on Euthanasia. This was performed by the IP injection of an FDA approved euthanasia solution (Beuthanasia-D Special, Merck Animal Health, Madison, NJ) in accordance with the manufacturer’s instructions based on the weight of the rat. Deep anesthesia was confirmed prior to a terminal cardiac bleed being performed using a 14-gauge needle and 10 mL syringe, with blood collected into both EDTA and serum separator tubes. Blood for CBC and serum chemistries were run the same day and the remaining serum stored at − 80 °C prior to use. The post caval lobe of the right lung, left lateral lobe of the liver, right kidney, and vastus lateralis and biceps femoris immediately adjacent to the femur were collected. Lung, liver, kidney tissue and skeletal muscle were then prepared and stored for histology, preparation protein lysates from tissues, and gene transcript expression analysis.

### Histology

Tissues were prepared in 10% neutral buffered formalin prior to paraffin embedding and sectioning at 5 μm. Tissue sections were then prepared and stained with hematoxylin and eosin (H&E). A board-certified veterinary pathologist, blinded to group and injury pattern examined the slides using a light microscopy under both low-power (2x) and high-power field of view (40 ×) (BX-41, Olympus America Inc., Center Valley, PA) Criteria for assessment included are shown below. Each criterion was assigned a numeric score ranging from 0 to 5 using the following grading scheme: normal (0), minimal (1), mild (2), moderate (3), marked (4), or severe (5). Scores for each criterion were summed to obtain a cumulative histopathology score for each section examined and assessed by each criterion between time point and injury pattern.

**Table:** 

Kidney	Liver	Muscle
Tubular necrosis/apoptosis, cortex	Hepatocyte apoptosis	Cellular infiltrate, mononuclear
Tubular necrosis/apoptosis, medulla	Hepatocyte necrosis	Cellular infiltrate, mixed
Tubular proteinosis	Portal hepatocyte radiating	Edema
Cellular infiltrate	Hepatic cord dissociation	Hemorrhage
Tubular basophilia	Increased hepatocyte mitosis	Fibroplasia
	Increased multinucleate hepatocyte	Perivascular inflammation
	Lipid change	Myocyte degeneration/ necrosis
	Extramedullary hematopoiesis (EMH)	

### Quantitative reverse transcription PCR (RT-qPCR) gene analysis

Tissue from the lung, kidney, liver, and skeletal muscle within the ZOI surrounding the amputation site were collected (tissue samples ≤ 0.5 cm in any direction) and stored in 1 mL of RNAlater™ stabilization solution (Invitrogen, Waltham, MA), initially at 4 °C for 72 h to allow full penetration of the solution through the tissues, before removing the tissue from the RNAlater™ solution and transferring to − 80 °C storage prior to RNA extraction. RNA was extracted using RNeasy Lipid Tissue Mini kits and protocols (Qiagen, Germantown, MD). Samples were weighed and approximately 90 mg of muscle tissue was homogenized using an Omni Tissue homogenizer (TH) with disposable hard tissue Omni Tip™ plastic homogenizing probes (Omni International, Kennesaw, GA) in 1 mL of QIAzol lysis reagent (Qiagen, Germantown, MD). RNA was extracted according to the manufacturer’s standards protocol. Complementary DNA (cDNA) reactions were performed using the iScript Advanced cDNA Synthesis Kit for RT-qPCR (Bio-Rad, Hercules, CA) following the manufacturer’s instructions. RT-qPCR was performed using a SYBR Green supermix (SsoAdvanced™ Universal SYBR Green Supermix, Bio-Rad, Hercules, CA) according to the manufacturer’s instruction. A 384-well custom array (4 samples per plate) was designed containing 87 gene targets covering a mixture of individual osteogenic, chondrogenic, and angiogenic genes, in addition to 5 normalization genes, *Gapdh*, *B2m*, *Actb*, *Hprt1*, *Rplp0,* and controls (Bio-Rad, Hercules, CA) for the assessment of early heterotopic ossification in injured muscle. The plates were optimized for use with the QuantStudio real-time PCR System (QuantStudio 7 Flex, Applied Biosystems, Waltham, MA). The thermal cycling protocol for the reaction commenced with a first stage of 90 s at 50 °C, followed by 10 min at 95 °C. The second stage consisted of 40 amplification cycles of 15 s denaturation at 95 °C, followed by 30 s at 60 °C for annealing and extension. The final stage consisted of a melt curve analysis was then performed at the end of the protocol between 60–95 °C using 0.5 °C increments at 2–5 s/step. Data was analyzed using Qbase + data analysis software, version 3.2 (Biogazelle, Zwijnaarde, Belgium), normalized against reference genes [43]. Data exclusion criteria included wells with a threshold cycle (Ct) of > 36. Normalization factor, fraction of detected targets and mean Ct value, were reviewed as part of sample quality control to identify erroneous sample runs. Statistical analysis was performed comparing the mean of gene expressions using the unpaired t-test with two-sided significance.

### Quantification of protein expression in tissue extracts

For tissue protein lysate analysis, the vastus lateralis and biceps femoris immediately adjacent to the femur and/or zone of injury was collected. Tissues were placed in placed in cryotubes and immediately flash frozen in liquid nitrogen prior to long-term storage at − 80 °C. Three genes identified as being differentially expressed on RT-qPCR in injured muscle when comparing tourniquet and non-tourniquet amputation groups were selected for quantification. Muscle samples obtained at POD 1, 3 and 7 were extracted. Protein concentration of the extract was determined using a BCA protein assay (cat# 23225, Pierce Biotechnology, Rockford, IL). Quantification of protein content in the tissue extracts were determined by ELISAs for runt-related transcription factor 2 (RUNX2), platelet-derived growth factor subunit A (PDGFA), (cat# LS-F53973, LS-F519, LifeSpan BioSciences, Seattle, WA) and integrin binding sialoprotein (IBSP) (cat# MBp2-70022, Novus Biologicals, Centennial, CO) following the manufacturer’s recommended instructions.

### Comprehensive metabolic panel (CMP)

Blood chemistry panels (Chem19) included a renal and electrolyte panel (BUN, Creat, Na+, K+, Cl−, CO_2_, Phos, Ca^2+^), a liver panel (AST, ALT, LDH, ALP, BILI) and a metabolic panel (Albumin, Total protein, Glucose, Cholesterol, Triglycerides) and creatine kinase (CK) and were performed on a Vitros 350 Chemistry System (Ortho-Clinical Diagnostics, Raritan, NJ).

### Markers of organ injury and systemic inflammation

Analysis of neutrophil gelatinase-associated lipocalin (NGAL) (cat# ab119602, ab239422, Abcam, Boston, MA) and TREM1 (MTRM10, R&D Systems, Minneapolis, MA) were performed using serum ELISA kits according to manufacturer’s instructions. HMGB1 and Calprotectin (S100A8/S100A9) (cat# LS-F11640, LSF22540, LifeSpan BioSciences, Seattle, WA) assays were performed on plasma samples according to the manufacturer’s instructions. Relative O.D. values were calculated and used to generate 4-PL standard curves to interpolate the concentrations of the samples, which were then adjusted by the appropriate dilution factors. The lower lobe of the right lung was used to calculate the wet-to-dry ratio of the lung. Following excision, the lower lobe was blotted to remove any excess blood or fluid from the outside of the lung. It was then weighed, and the wet weight of the lung calculated. The lung was then placed in a drying oven at 60 °C and weighed daily until the weight stabilized across at least a 24-h period, and the ratio of the weights calculated. The assessment of circulating immune mediators in serum samples was conducted using Bio-Plex Pro™ Rat Cytokine 23-Plex assays (cat# 12005641, Bio-Rad, Hercules, CA) performed on a Bio-Plex 200 Luminex system with high throughput fluidics (cat# 171000205, Bio-Rad, Hercules, CA) which had undergone both validation (Bio-Plex Validation Kit 4.0, Hercules, CA) and daily calibration prior to use (Bio-Plex Calibration Kit, Hercules, CA) to standardize signal output and sensitivity. Assay working ranges were determined using Bio-Plex Manager 6.1 software (Bio-Rad, Hercules, CA) and used for the automatic identification and removal of outliers and optimization of 5-PL standard curves to interpolate the concentration of each analyte.

### Statistical analysis

The criteria for statistical significance were defined as *p* < 0.05, and tests were performed for two-sided significance. Data was assessed for gaussian distribution using D’Agostino-Pearson normality testing, or Shapiro–Wilk testing for smaller sample size, with outlier analysis performed using the ROUT method. Parametric data is presented as a mean with 95% confidence intervals (95% CI) unless otherwise stated. Nonparametric data is presented as a median and inter-quartile range (IQR). Analysis was performed using either an unpaired t-test for parametric data, or the Mann–Whitney test for nonparametric data. Multiple variables with Gaussian distribution were compared using either one-way or two-way ANOVA, with post hoc testing performed on preselected pairs and corrected for multiple comparisons where indicated. Gene expression analysis was performed using Qbase + data analysis software, version 3.2 (Biogazelle, Zwijnaarde, Belgium), with results reported as mean values with standard error of the mean (SEM) based on an unpaired t-test. The inter-rater reliability of bone volume measurements across raters was determined using the intra-class correlation coefficient (ICC). Statistical analysis was performed except for gene expression data using Prism 8.1.2 for Mac (GraphPad Software, La Jolla, CA) and SPSS 25 for Mac (IBM Corp, Armonk, NY) software.

## Results

### Tourniquet use increases ectopic bone and elevated irregular periosteal reaction in a model of blast associated complex lower limb traumatic amputation

**Fig. 1:**
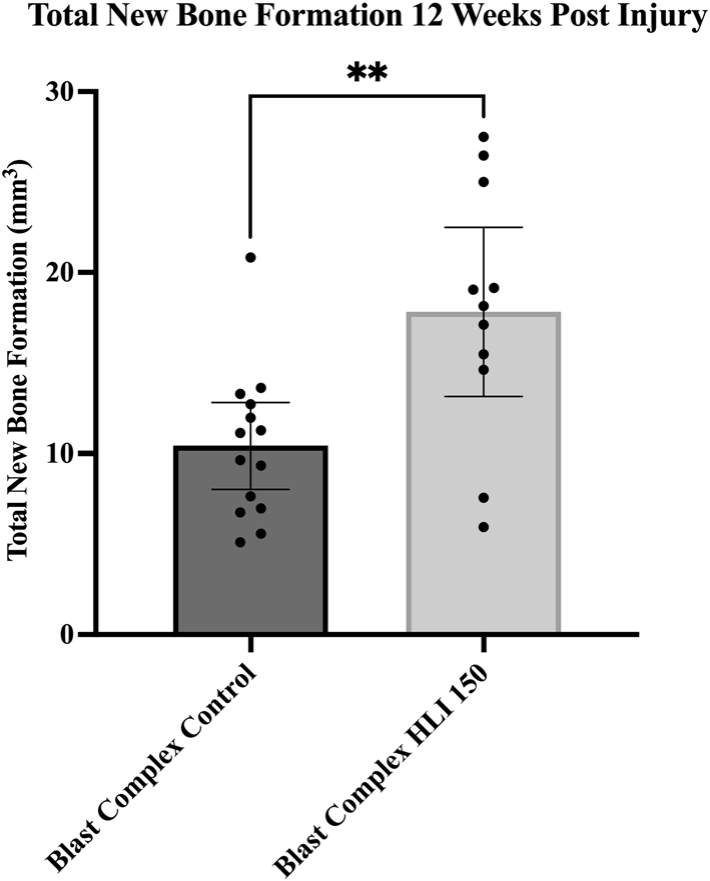
Total new bone formation 12 weeks post injury. Rats were subjected to a blast injury model with whole-body exposure to blast overpressure, followed by controlled femoral fracture, soft tissue quadriceps crush injury, with 150 min of tourniquet use (Blast Complex HLI 150) or without tourniquet use (Blast Complex Control), and limb amputation through the zone of injury. Micro-computed tomography scans were performed on animals following blast injury with or without tourniquet use at 12 weeks. Total new bone was determined by defining the difference between new bone and naïve bone. Results graphed as mean and 95% CI. Statistical comparison performed using unpaired t-test with Welch’s correction. *p < 0.05; **p < 0.01, ***p < 0.001, ns not significant

Non-invasive µCT imaging was used to determine whether tourniquet application increases the volume of ectopic bone formed and leads to elevated irregular periosteal reaction in our model of complex lower limb trauma. Volumetric assessment with μCT scans (*n* = 11) performed at 12 weeks post injury demonstrated a 70% increase in total new bone formation in the Blast Complex HLI 150 tourniquet group compared to μCT scans in the Blast Complex Control group (*n* = 14) (17.8 ± 7.0 mm^3^ (95% CI 13.1–22.5 mm^3^) vs 10.4 ± 4.1 mm^3^ (95% CI 8.0–12.8 mm^3^), mean ± SD, *p* = 0.007) (Fig. 1). Whole limb sectioning and H&E staining was performed to confirm that μCT findings were consistent with histological evidence of chondro-osteogenesis (data not shown).

### Early upregulation and prolonged expression of inflammatory mediators following tourniquet use

**Fig. 2:**
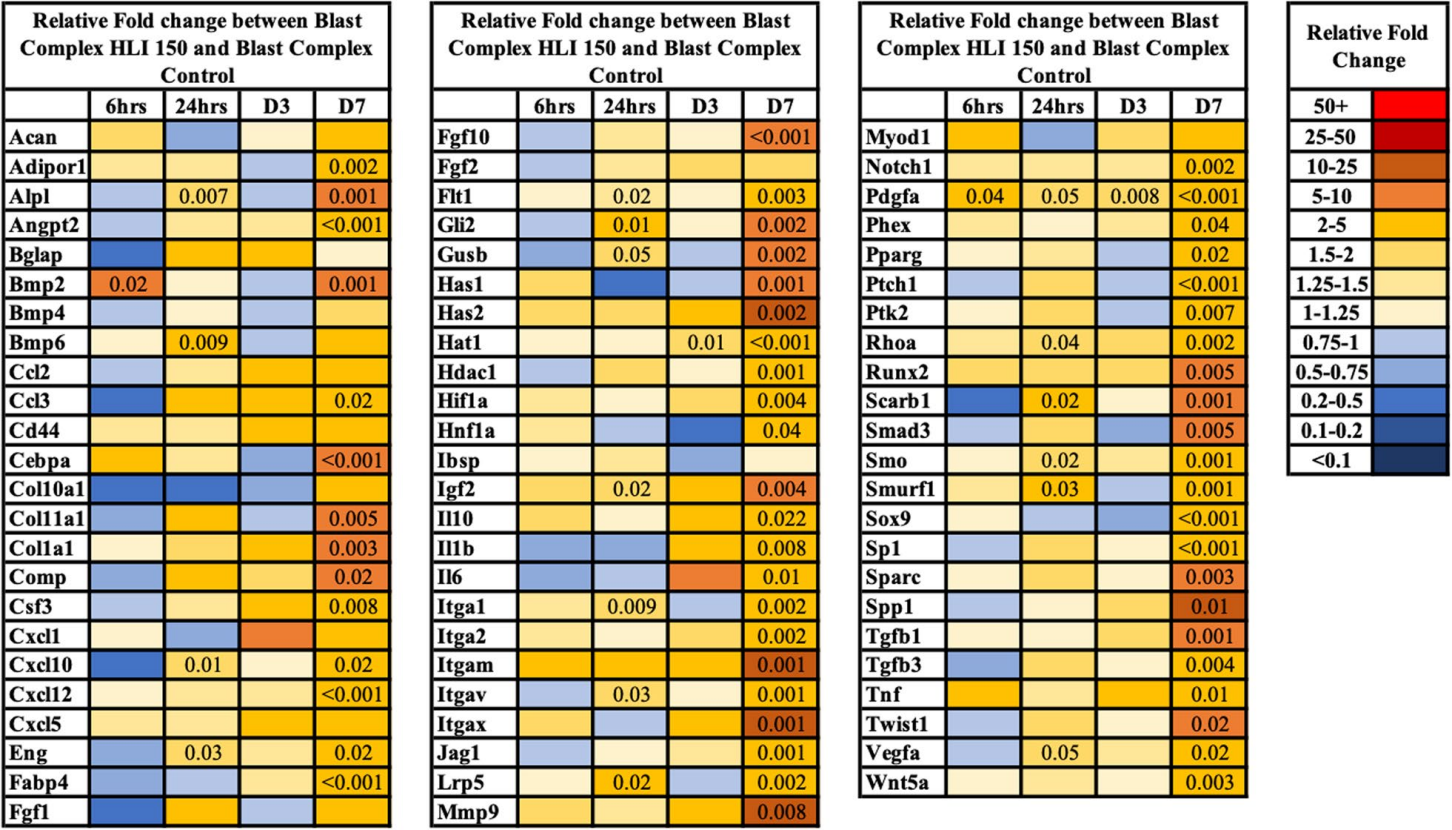
Expression of chondrogenic, angiogenic and osteogenic genes associated with early formation of traumatic heterotopic ossification. Relative fold change of gene expression between Blast Complex HLI 150 and Blast Complex Control, defined as the ratio of the mean of Blast Complex HLI 150 group gene expression compared to mean Blast Complex Control group gene expression, where both gene expression fold changes have been normalised to naïve tissue expression prior. Statistical analysis performed using unpaired t-test. Only comparisons reaching statistical significance shown

We first aimed to quantify the cellular response to tourniquet induced ischemia following extremity trauma through measurement of muscle tissue *Hif1α*, inflammatory mediators, and early osteo-chondrogenic gene expression patterns between the two groups from 6 h to POD 7. *Hif1α* expression was upregulated in the Blast Complex HLI 150 tourniquet group relative to Blast Complex Control group by 1.42-fold at 6 h to 4.03-fold higher at POD 7 (*p* = 0.004). Tissue gene transcripts for inflammatory chemokines and cytokines *Ccl2*, *Ccl3*, *Cxcl1*, *Cxcl5, Cxcl10, Cxcl12*, *Csf*3, *Il1β*, *Il6* and *Tnfα* demonstrated early and prolonged expression in the tourniquet treated group from 24 h to 7 days in the tourniquet group (Fig. 2). *Pdgfa*, an early mesenchymal stem/progenitor growth factor, was the most consistently upregulated gene in the tourniquet group compared to the control group during the first 7 days (*p* < 0.001–0.05) (Fig. 2). Tissue transcript levels of *Eng,* a key protein of angiogenesis and vascular endothelium, was 2.0-fold higher in the tourniquet group at 24 h (2.02 vs 1.02, *p* = 0.03) to 4.4-fold higher than the control group at POD 7 (2.95 vs 0.54, *p* = 0.02). These findings support our hypothesis that tourniquet use leads to greater and prolonged expression of the hypoxia transcription factor *Hif1α* in blast complex limb injury model, with upregulation and persisting expression of inflammatory, angiogenic and early mesenchymal stem/progenitor growth factors which are associated with heterotopic ossification formation [1, 27, 28, 44–46].

### Tourniquet-induced skeletal muscle ischemia and muscle damage upregulates genes encoding for extracellular matrix, innate immune effector cell adherence, and osteogenesis

Transcripts for receptors and molecules involved in innate cell adhesion, leukocyte extravasation (*Itgam, Itga1, Itga2, CD44*), and extracellular matrix proteins (*Has2*), which allow cells to migrate and provide a framework for ingrowth of blood vessels were increased in the Blast Complex HLI 150 tourniquet group compared to the Blast Complex Control group across all measured time points (Fig. 2). *Itgam, Itga1, Itga2, CD44,* and *Has2* were increased 2.1–21.1-fold, 1.4–3.7-fold, 1.1–4.5-fold, 1.4–2.3-fold, and 1.7–10.8-fold, respectively, in the tourniquet group compared to the control group. These findings suggest that tourniquet use results in early expression of factors that increase inflammation and angiogenesis.

At 6 h after injury, bone morphogenetic protein (BMP) signaling was upregulated in the Blast Complex HLI 150 tourniquet group compared to the control group, with greater response and more prolonged gene expression of *Bmp2, Bmp4* and *Bmp6*. Most notably, *Bmp2* expression was increased 6.1-fold at 6 h post injury (*p* = 0.02) and 6.7-fold at POD 7 (*p* = 0.001) compared to the Blast Complex Control group (Fig. 2). *Bmp6* expression was increased to 2.3-fold on POD 1 (*p* = 0.009). Gene transcript expression for osteoblast differentiation (*Runx2*, 1.6–7.7-fold; *Spp1*, 0.8–18.2-fold, *Bglap*, 0.5–4.5-fold), bone extracellular matrix protein (*Mmp9*, 1.4–16.7-fold), and RhoA/ROCK signaling (*Rhoa*, 1.4–3.0-fold) were all higher in the tourniquet group compared to complex control group (Fig. 2). Together, these results indicate that tourniquet induced ischemia leads to alterations in osteogenic signaling that increase bone formation.

### Tourniquet use increased protein expression of pro-osteogenic factors following a blast combat applicable model of traumatic lower limb amputation

**Fig. 3:**
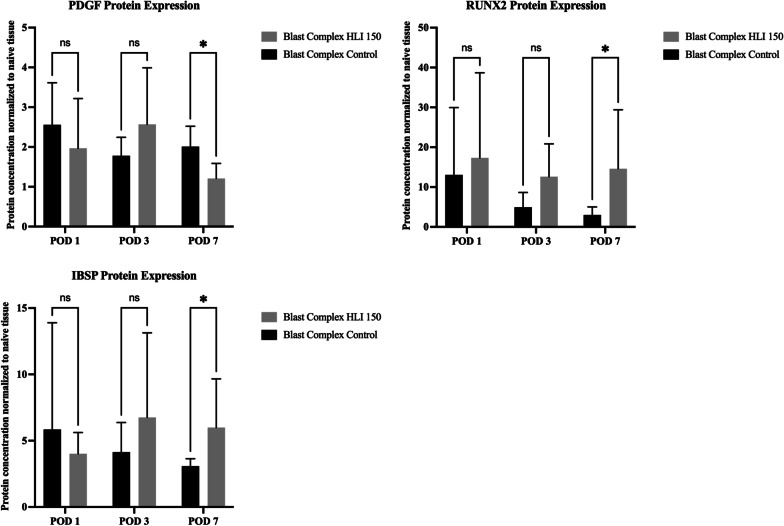
Protein expression in injured muscle homogenates is consistent with upregulated mRNA of associated genes. Graphs show comparison of the protein expression of PDGF, RUNX2, and IBSP between Blast Complex HLI 150 and Blast Complex Control at Post-Operative Day (POD) 1–7 following injury. Protein concentrations were normalized to naïve tissue expression and adjusted for total injured muscle protein levels. Graphs shown as mean and 95% CI. Statistical comparison performed using two-way ANOVA. *p < 0.05; **p < 0.01; ***p < 0.001, ns not significant

Three of the genes identified as being differentially expressed at the RNA level in the tourniquet and control groups were selected for protein quantification by ELISA. Integrin Binding Sialoprotein (IBSP), a mineralized protein which forms part of the bone matrix and is present during initial bone formation, trended toward higher expression from POD 3, reaching significance on POD 7 (1.1 vs 0.5 ng/mg, *p* = 0.03; Fig. 3). RUNX2, a master transcription factor for osteogenesis, was elevated on POD 7 (269.8 vs 44.2 ng/mg, *p* = 0.03), with higher mean expression present as early as POD 1 in the Blast Complex HLI 150 tourniquet group (Fig. 3). PDGFA expression showed no consistent pattern of elevation, with an elevation in the Blast Complex HLI 150 tourniquet group on POD 1 (0.2 vs 0.1 ng/mg, *p* = 0.04); however, the Blast Complex Control group demonstrated higher expression of PDGF expression by POD 7 (0.08 vs 0.14 ng/mg, *p* = 0.02; Fig. 3). These results suggest that the changes seen in osteogenic gene transcript expression translates to increased protein expression (Fig. 3).

### Addition of tourniquet application to a complex injured limb results in a heightened and prolonged systemic inflammatory response

**Fig. 4:**
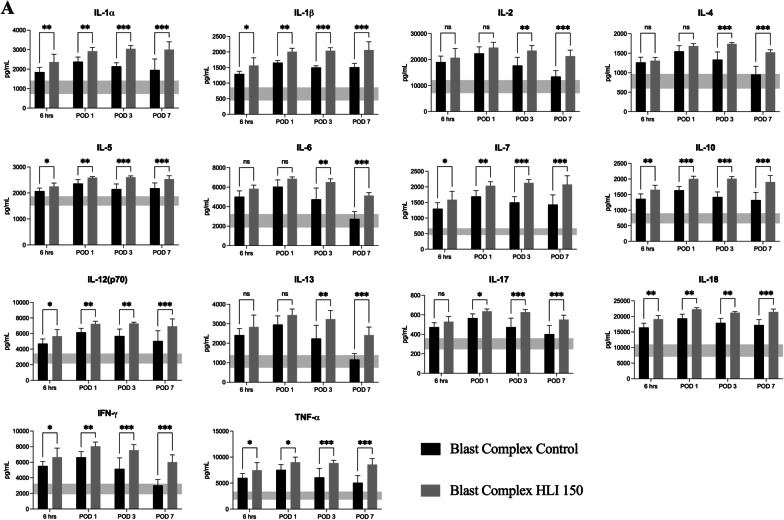

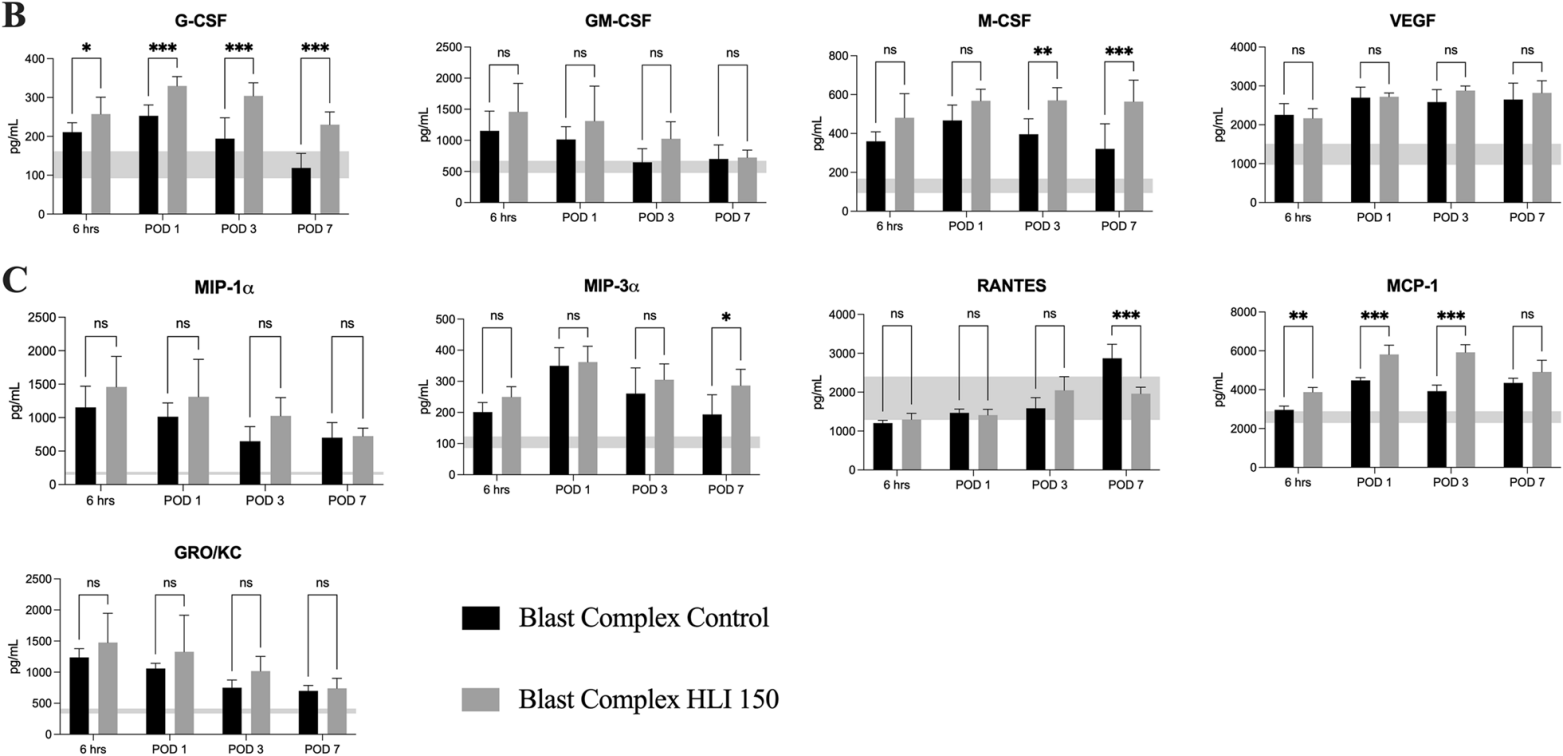
Elevated and prolonged systemic inflammatory response occurs following tourniquet application to a complex injured limb. Graphs show serum concentrations of **A** cytokines, **B** chemokines, and **C** growth factors between 6 h and Day 7 post injury in the Blast Complex Control and Blast Complex HLI 150-min groups. Graphs shown as mean and 95% CI with statistical testing using two-way ANOVA with Holm-Sidak corrected for multiple comparisons. Shaded horizontal band denotes the 95% CI of the mean serum concentrations of analytes in the naïve group. Significance shown as *p < 0.05; **p < 0.01; ***p < 0.001, ns not significant

Multiple inflammatory mediators in the serum were assessed from 6 h to POD 7. At 6 h post injury, the Blast Complex HLI 150 tourniquet group exhibited statistically significant higher serum concentrations of IL1α, IL1β, IL5, IL7, IL10, IL12(p70), IL18, G-CSF IFNγ, TNFα and MCP-1 (*p* = 0.003–0.05; Fig. 4). On POD 1, IL-17 plus all those significantly elevated at 6 h remained significantly increased in the tourniquet group compared to the non-tourniquet group (*p* ≤ 0.001–0.01; Fig. 4). By POD 3, elevations in IL2, IL4, IL6, IL13 and M-CSF also reached significance (*p* = 0.002–0.005; Fig. 4). At POD 7, MIP-3α, in addition to all the previously listed analytes were statistically elevated in the tourniquet group (*p* = 0.004–0.05; Fig. 4). Elevated expression of MCP-1 persisted but were no longer significant between groups (*p* = 0.06) and RANTES showed greater expression in the control group than the tourniquet group (*p* < 0.001). Elevated expression of GM-CSF, GRO/KC, MIP-1α*,* and VEGFA were noted in the tourniquet group at all timepoints, but these didn’t reach significance between the injured groups. Collectively this inflammatory mediator panel data shows that tourniquet use leads to a prolonged and sustained systemic inflammatory response compared to blast complex limb injury in isolation. Elevated serum levels of these pro-inflammatory cytokines (IL6, IL10, IL13, IFNγ, MCP-1) have been demonstrated to be associated with heterotopic ossification formation in humans post injury [47, 48]. Additionally, these findings demonstrate a positive correlation between systemic circulating levels of pro-inflammatory mediators and gene transcript expression locally at the site of injury as described earlier.

### Tourniquet use results in biochemical and histological evidence of remote organ injury

**Fig. 5:**
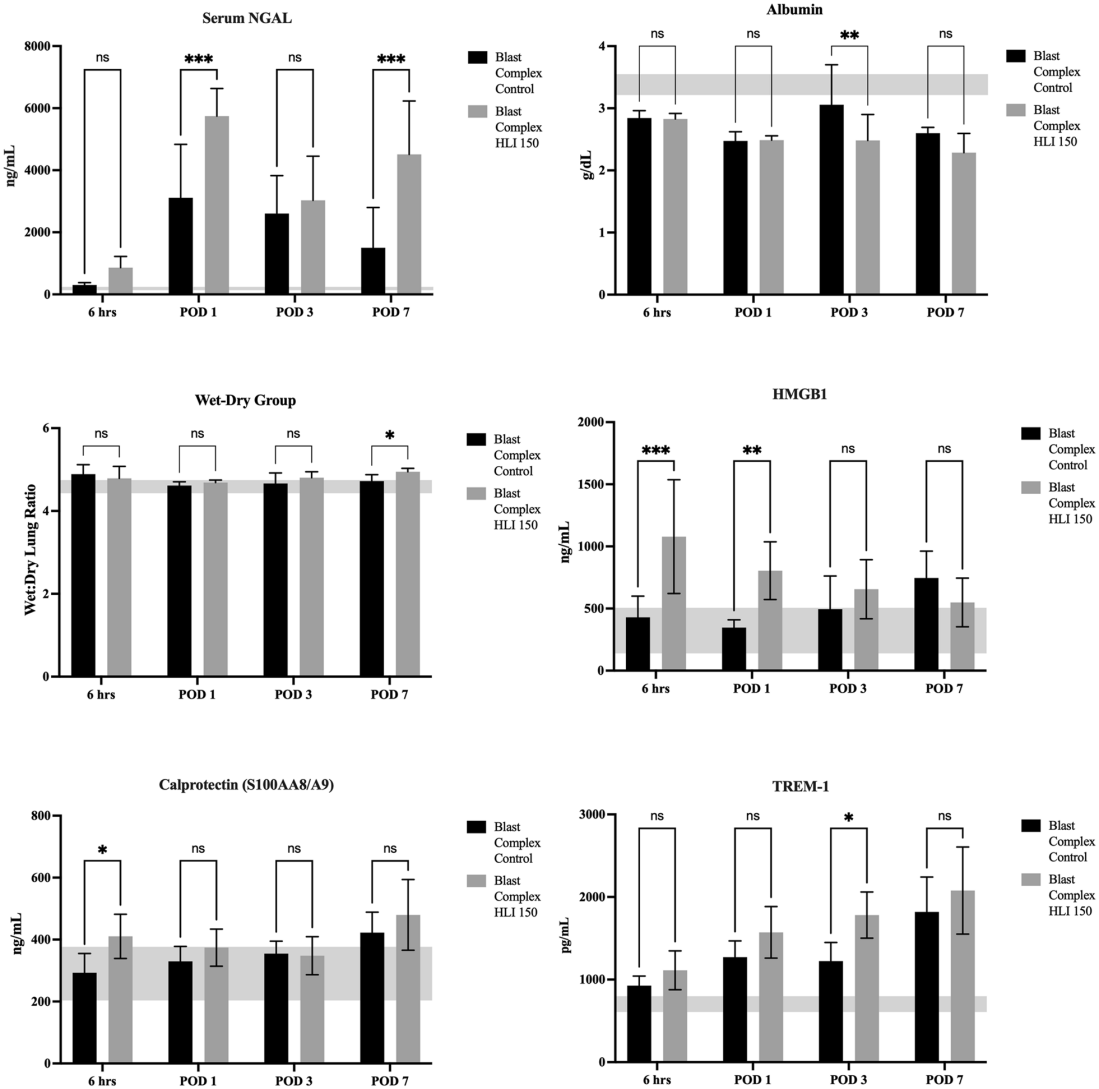
Tourniquet use leads to increased evidence of remote organ injury. Graphs show comparison of NGAL, Albumin, Wet:Dry Lung Ratio, HMGB1, Calprotectin, and TREM-1, between Blast Complex HLI 150 and Blast Complex Control from 6 h to POD 7 following injury. Graphs shown as mean and 95% CI with statistical testing using two-way ANOVA with Holm-Sidak correction for multiple paired comparisons where appropriate. Shaded horizontal band denotes the 95% CI of the mean serum concentrations of analytes in the naïve group. Significance shown as *p < 0.05; **p < 0.01; ***p < 0.001, ns not significant

Analysis of biochemical markers was performed to evaluate the additional effects of tourniquet use in the blast injury model on end organ injury. Analysis of renal injury using serum urea and creatinine showed that while the Blast Complex HLI 150 tourniquet group had higher mean concentrations from 6 h to POD 7, these failed to reach significance between injured groups. Creatinine was only significantly elevated above naïve baseline in the tourniquet group, occurring at 6 h post injury (0.8 vs 0.5, *p* = 0.04), while the non-tourniquet group experienced no similar rise. Serum creatinine is recognized as a late and often inadequate marker of acute kidney injury (AKI), while neutrophil gelatinase-associated lipocalin (NGAL), an established biomarker of AKI may detect AKI up to 48 h earlier than traditional criteria [49]. Analysis of serum NGAL in these models demonstrated a clear increase in renal injury with the addition of tourniquet use. NGAL was increased at all time points in the Blast Complex HLI 150 tourniquet group compared to the Blast Complex Control group, reaching significance at POD 1 and 7 (*p* < 0.001) (Fig. 5). Renal function is recognized to be associated with impaired bone healing, with chronic kidney disease shown to negatively affect bone regeneration in rat models [50], and even an AKI which resolves can have long term effects on bone health [51].

Hepatocellular injury was next assessed using ALT, AST, and albumin, with the greater rises seen in serum ALT and AST at 6 h post-injury in Blast Complex HLI 150 tourniquet group (186.0 U/L; 499.9 U/L respectively) compared to the Blast Complex Control group (110.6 U/mL; 403.0 U/L) but failed to reach significance between injured groups (*p* = 0.19 and *p* = 0.29). By POD 1, ALT and AST expression between the two groups had equalized, although both remained significantly elevated compared to naïve baseline (*p* < 0.001). Albumin levels fell across all injured groups compared to naïve baseline following injury. Reductions were greater in the tourniquet group, reaching significance between the two injured groups at POD 3 (*p* = 0.008), while significance at POD 7 fell just short once multiple comparisons adjustments were included (Fig. 5). This data suggests that tourniquet use may result in an earlier, more accelerated hepatocellular injury in a traumatic blast amputation model. Hypoalbuminemia is a recognized risk factor for delayed wound or bone healing following surgery and injury. Impaired or chronic liver dysfunction can also alter bone growth as liver enzymes can affect bone growth and loss through factors such as IGF-1, IL6, Vitamin D and OPG [52–54].

Evidence of acute lung injury was assessed by analysis of the wet-to-dry ratio (wet:dry) of lung tissue, to assess for increases in lung permeability and alveolar epithelial dysfunction. From 6 h post injury, the lung wet:dry ratio increased in the Blast Complex HLI 150 tourniquet group compared to the Blast Complex Control group,with inter-group significance reached at POD 7 (4.961 vs 4.804, *p* = 0.02) (Fig. 5).

To establish if tourniquet use resulted in increased markers of cell inflammation and tissue injury, assays to detect circulating damage associated molecular patterns (DAMPs) biomarkers HMGB1, TREM1 and Calprotectin (S100A8/A9) were performed. All three analytes showed increased expression in the Blast Complex HLI 150 tourniquet group. At 6 h and day 1 post injury, HMGB1 was significantly elevated compared in the tourniquet group compared to Blast Complex Control group (1078.4 ± 495.2 vs 429.6 ± 162.1 ng/mL, *p* < 0.001 and 804.5 ± 302.4 vs 347.0 ± 67.7 ng/mL, *p* = 0.006) (Fig. 5). This nuclear DNA binding protein, once in the extracellular compartment following cell injury, has a pro-inflammatory effect and in humans, plasma levels correlate to degree of organ dysfunction and outcome. TREM-1 is an innate immune receptor, whose inhibition has been shown to reduce inflammation and oxidative stress. Serum calprotectin, another marker of inflammation, was also elevated with tourniquet use, most significantly in the early timeframe at 6 h (410.4 ± 77.2 vs 293.0 vs 59.2 ng/mL, *p* = 0.04) (Fig. 5). The tourniquet group had increased expression of TREM-1 at all time points, with significance reached at POD 3 (1781.1 ± 463.7 vs 1223.8 ± 244.0 pg/mL, *p* = 0.02) (Fig. 5). Combined, these biomarkers further support that tourniquet use increases systemic inflammation and subsequent end organ dysfunction compared to a blast complex limb injury alone.

**Fig. 6:**
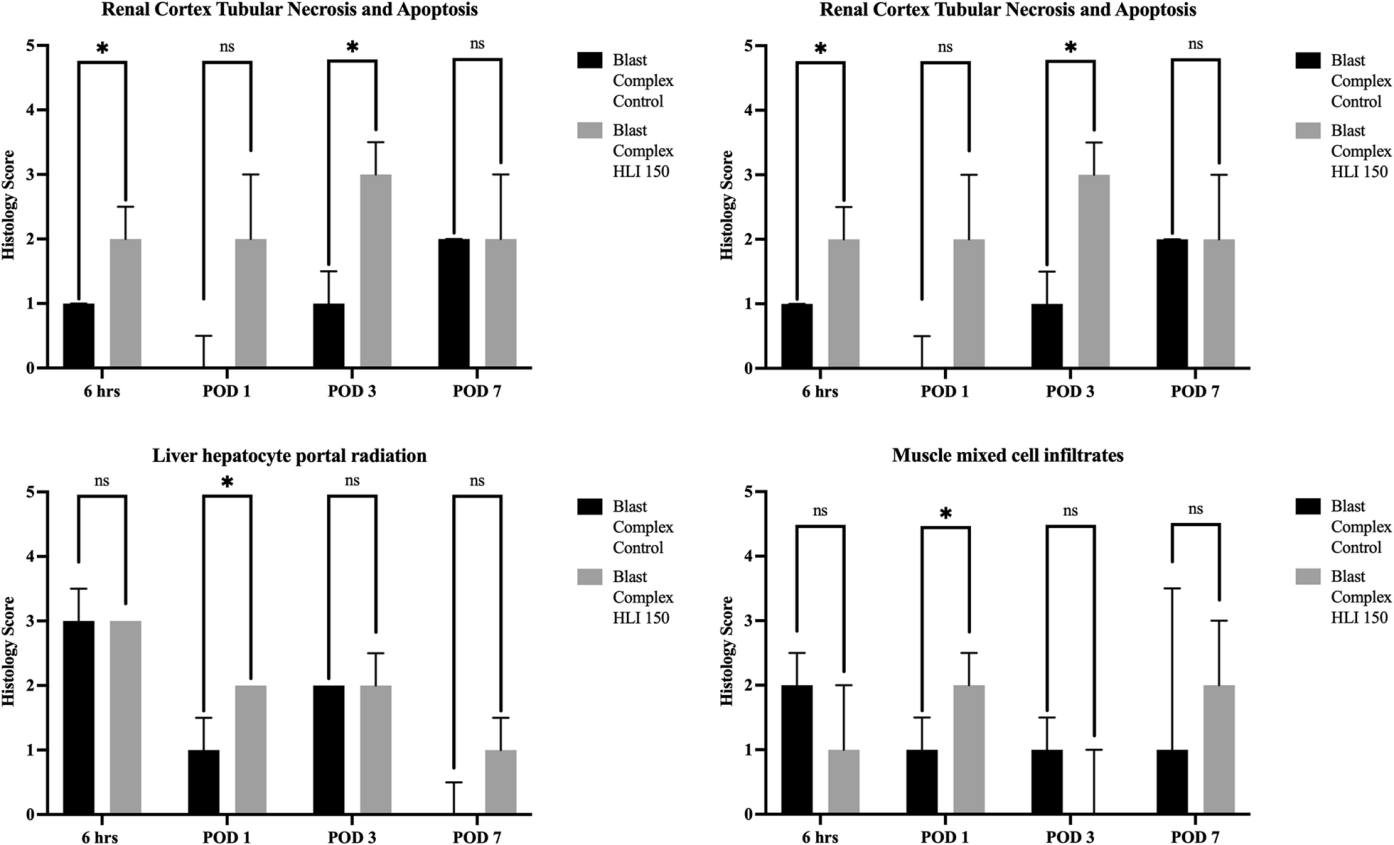
End organ histological findings. Histological scoring was performed by a veterinary pathologist blinded to group and timing. Liver, Kidney and Muscle were assessed using a standardized categorical scoring system from 0–5 for each sample. Graphs show Renal Cortex, Renal Medulla, Liver and Muscle. Median and IQR range plotted with statistical analysis performed using Mann–Whitney test for a pairwise comparison. Significance shown as *p < 0.05; **p < 0.01; ***p < 0.001, ns not significant

Histological examination of renal tissue following tourniquet application identified increased evidence of tubular necrosis and apoptosis in both the renal cortex and medulla (Fig. 6). Renal cortex injury was the most consistent injury between 6 h and POD 3, and similarly with the renal medulla. The outer renal medulla is an area sensitive to hypoxic injury, and therefore apoptosis and necrosis would be expected to occur, with changes more commonly seen in the early 6 h samples suggesting acute injury and cell death. Within the liver, changes histologically were less pronounced, with only liver hepatocyte radiation reaching significance (Fig. 6). For injured muscle, cellular infiltrates and edema were the most variable categories with only mixed cellular infiltrates reaching significance (Fig. 6) were the most different between injured groups. Histological findings were consistent with the evidence of biochemical dysfunction described earlier with renal tissue most sensitive to hypoxic injury and evidence of additional acute injury beyond that exhibited in the blast complex injury model.

## Discussion

As high as 90 percent of combat blast-associated traumatic amputations develop HO [9]. While hypoxia caused by tourniquet use has been theorized to increase HO formation, no studies have been performed previously [25]. By assessing the effects of tourniquet application in the development of chondro-osteogenesis and tHO in this study, we sought to further understand the influence of IRI on the development and exacerbation of this debilitating condition. Using in vivo μCT imaging, we detected an increase in ectopic bone formation in injured limbs subjected to 150 min of tourniquet prior to amputation compared to no tourniquet application. Tourniquet use and IRI can exacerbate and prolong the inflammatory responses and activate pro-osteogenic pathways. Thus, our study provides evidence that supports the theory that increased osteogenesis occurs following blast injury and tourniquet application, contributing to tHO formation.

The molecular pathways of early tHO formation are complicated and have not yet been fully elicited. The role of hypoxia in aberrant bone deposition has been well described [44, 55, 56], with HIF-1α situated at the molecular crossroads between both hypoxic and inflammatory signaling [28, 57–59]. HIF-1α signaling has been shown to affect neutrophil and macrophage function by controlling pro-inflammatory gene signaling, upregulate phagocytic activity, and reduce neutrophil apoptosis [60]. Additionally, HIF-1α signaling in mesenchymal progenitor cells (MPCs) has been shown to drive aberrant osteochondral differentiation in HO [28]. Our gene expression data demonstrated that *Hif1a* expression remained consistently upregulated for 7 days in the tourniquet group compared to the control group. These findings indicate that even with the majority of ischemic tissue removed by amputation, tourniquet application resulted in greater and prolonged hypoxia in the zone of injury. *Pdgfa*, a gene which encodes for a protein known to be associated with HO formation and fibrosis [61, 62], was found to be the most consistently upregulated gene across the time course of this study. Given the critical importance of angiogenesis in the recruitment of MPCs, which are chondro-angio-osteogenic progenitor cells involved in early HO formation, the significant upregulation of these genes in the tourniquet applied group further supports the observational findings and highlights some of the molecular mechanisms which we sought to investigate. Both PDGF and HIF-1α inhibitors have been shown to significantly reduce HO volume in rodent Achilles tenotomy models [28, 61, 63], suggesting that these therapies may also mitigate the angiogenic effects of prolonged tourniquet use in tHO development.

Local and systemic inflammation have been shown clinically to be key drivers of tHO, specifically the expression of IL3, IL6, IL10, IL12(p70), IL10, CXCL10, MIP-1α (CCL3) and MCP-1 (CCL2) [47, 48]. Our gene expression data demonstrates prolonged and upregulated expression of many of the genes associated with these proteins in the tourniquet use group compared to control group. Our inflammatory mediator analysis has shown that tourniquet use in this model increases DAMP expression, and causes an exaggerated and sustained elevation of systemic inflammatory mediators over the time course of the study including IL1α, IL1β, ILl6, IL13, IL18, G-CSF, IFNγ, TNF-α and MCP-1*.* Combined with biochemical and histological data of end organ dysfunction, these findings support our initial hypothesis that tourniquet use results in a more exaggerated and persistent pro-inflammatory state that ultimately leads to continued tissue hypoxia, cellular apoptosis and necrosis, and exaggerated tissue injury following IRI. Furthermore, BMPs have also been shown to regulate endochondral ossification by promoting cell proliferation and differentiation of prechondrogenic cells, with their role in HO well described [64, 65]. Early upregulation of BMPs with tourniquet use compared to control was observed as early as 6 h post-injury, priming the tissue environment for osteogenesis [64]. Osteoblastic differentiation is controlled by a range of factors that influence bone formation. RUNX2 is involved in osteoblast differentiation from precursors by acting as a key transcription factor to produce osteoblasts and is downstream of BMP and WNT signaling. Key downstream genes of RUNX2 involved in osteoblast differentiation were all elevated in the tourniquet group (*Bglap, Ibsp, Mmp9, Spp1*) based on our qRT-PCR, demonstrating a clear upregulation of this pathway consistent with the increased HO formation observed with the μCT findings. This data supports that tourniquet induced skeletal muscle damage manifested by limb hypoxia, IRI and increased inflammation, further upregulates early key transcription factors and signaling molecules involved in early endochondral ectopic bone formation. An exaggerated and persistent systemic inflammatory response state was also demonstrated with evidence of remote organ biochemical dysfunction and injury beyond that seen in the blast complex injury model alone, confirming that a 2.5 h prolonged tourniquet application causes a significant additional injury burden in our model.

While the study design and models for this research were chosen to replicate the injury patterns that have become commonplace following blast injury in our service personnel, this study has several limitations to translation to clinical practice. Differences in inter-species size and scaling of injuries limits the generalizability of the duration effect to identify where a tourniquet duration threshold may be present in humans, only that the tourniquet effect is present and adds an additional injury burden in blast complex lower injury. Our current model does not include a hemorrhage phase prior to tourniquet application as would occur in trauma, and therefore renal effects from hypoperfusion are likely to be magnified by a real-world injury requiring tourniquet application, and subject to the increasingly common strategy of hypotensive resuscitation. While many of our results reached statistical significance, due to the increased biological variability encountered in a complex injury model, variance in results was often high affecting statistical calculations. Larger sample sizes for future studies should therefore be considered and could be countered with reduced time points to optimize study design. Further work is required to characterize the genomic and proteomic profile associated with blast injury associated tHO formation subject to ischemia reperfusion injury. Identification of these signalling pathways provide potential targets for early therapeutic intervention to reduce systemic or local tissue dysfunction. Characterizing complex injury in a large animal model of similar size to humans may allow a more representative analysis of the physiological and immunologic changes experienced and subsequent duration thresholds at which prolonged tourniquet use may have an additional deleterious effect on survival and tHO formation.

Prolonged field care (PFC) is recognized as one of the greatest capability gaps facing military forces. The development of models that replicate the complexity of blast limb trauma and seek to replicate the inflammatory milieu that arises provide a platform for pre-clinical investigation to enable bench-to-bedside-to-battlefield innovation. By further understanding this newly identified effect of blast on IRI, and thus how different combat injuries may affect safe tourniquet use, we seek to prevent avoidable IRI and secondary injury.

## Conclusions

This study demonstrates that in a rat model of blast-associated lower limb trauma and amputation, tourniquet application of 150 min increases tHO formation by 70% with an associated upregulation of angiogenic, inflammatory, and osteogenic genes associated with chondro-angio-osteogenesis, and an exaggerated and sustained systemic inflammatory response. Mechanism of injury and subsequent adjuncts in the early treatment of patients can affect the local and systemic inflammatory response leading to secondary injury. The lifesaving benefits of tourniquets with catastrophic hemorrhage remain clear and should continue to be championed both within the military and civilian environment. However, understanding the underlying mechanism and pathways activated by tourniquet use in promoting tHO formation will allow the development of therapeutic agents that minimize the aberrant osteo-chondrogenesis and other systemic effects. Prolonged field care seeks to reduce morbidity as well as mortality; this data supports the need to maintain minimal tourniquet times in blast associated trauma by reassessment of the injured limb and consideration of replacing a tourniquet with hemostatic dressings where clinically indicated, and by maintaining logistical and doctrinal timelines to early surgical intervention.

## Data Availability

The datasets used and/or analysed during the current study are available from the corresponding author on reasonable request.

## Abbreviations

AKI: Acute kidney injury
ALP: Alkaline phosphatase
ALT: Alanine transaminase
AST: Aspartate aminotransferase
BILI: Bilirubin
BMP: Bone morphogenetic protein
BUN: Blood urea nitrogen
CBC: Complete blood count
DAMPs: Damage associated molecular patterns
DNA: Deoxyribonucleic acid
HIF-1α: Hypoxia-inducible factor 1-alpha
HMGB1: High mobility group box protein 1
HO: Heterotopic ossification
IP: Intra-peritoneal
IRI: Ischemia reperfusion injury
LDH: Lactate dehydrogenase
MPC: Mesenchymal progenitor cell
NGAL: Neutrophil gelatinase-associated lipocalin
POD: Post-operative day
PFC: Prolonged field care
qRT-PCR: Quantitative reverse transcription polymerase chain reaction
RNA: Ribonucleic acid
tHO: Traumatic heterotopic ossification
TREM: Triggering receptor expressed on myeloid 1
μCT: Micro computed tomography
ZOI: Zone of injury
